# TonEBP expression is essential in the IL-1β–induced migration and invasion of human A549 lung cancer cells

**DOI:** 10.32604/or.2023.030690

**Published:** 2023-11-15

**Authors:** HEE JU SONG, TAEHEE KIM, HAN NA CHOI, SOO JIN KIM, SANG DO LEE

**Affiliations:** Department of Physiology, School of Medicine, Chungnam National University, Daejeon, 35015, Korea

**Keywords:** Lung cancer, TonEBP, Tumor microenvironment, Tumor-associated macrophage, IL-1β

## Abstract

Lung cancer has the highest mortality rate among all cancers, in part because it readily metastasizes. The tumor microenvironment, comprising blood vessels, fibroblasts, immune cells, and macrophages [including tumor-associated macrophages (TAMs)], is closely related to cancer cell growth, migration, and invasion. TAMs secrete several cytokines, including interleukin (IL)-1β, which participate in cancer migration and invasion. p21-activated kinase 1 (PAK1), an important signaling molecule, induces cell migration and invasion in several carcinomas. Tonicity-responsive enhancer-binding protein (TonEBP) is also known to participate in cancer cell growth, migration, and invasion. However, the mechanisms by which it increases lung cancer migration remain unclear. Therefore, in this study, we aimed to elucidate the mechanisms by which IL-1β and TonEBP affect lung cancer cell migration and invasion. We found that A549 cocultured-MΦ-secreted IL-1β induced A549 cell migration and invasion via the PAK1 pathway. TonEBP deficiency reduced A549 cell migration and invasion and increased responsiveness to IL-1β–induced migration and invasion. PAK1 phosphorylation, which was promoted by IL-1β, was reduced when TonEBP was depleted. These results suggest that TonEBP plays an important role in IL-1β induction and invasiveness of A549 cells via the PAK1 pathway. These findings could be valuable in identifying potential targets for lung cancer treatment.

## Introduction

Lung cancer has the highest mortality rate among all cancers [1–3], primarily because the early symptoms are insignificant, making diagnosis difficult, and because it rapidly develops drug resistance and readily metastasizes. Among the two types of lung cancer, non-small cell lung cancer (NSCLC) and small cell lung cancer [1,2], adenocarcinoma (an NSCLC), shows the highest mortality rate.

The tumor microenvironment (TME) is a major contributor of tumor progression or metastasis and consist of immune cells, blood vessels, fibroblasts, and macrophages that surround tumors [4–6]. In the TME, various cell types such as tumor-associated macrophages (TAMs), cancer associated fibroblasts (CAFs), and tumor endothelial cells (TECs) are found, and these cells induce cancer cell proliferation, migration, and angiogenesis [7]. It is well known that CAFs in the TME play an important role in forming tumors by reducing apoptosis and inducing proliferation and migration of cancer cells, whereas TECs are known to aggressively change cancer cells by supplying nutrients that induce tumor angiogenesis [7–9].

Cancer cells and macrophages (MΦ) share many cytokines and growth factors that promote cancer cell aggressiveness [10,11]. TAMs, which secrete several cytokines, chemokines, and growth factors [5,12], worsen cancer prognosis by inducing cancer cell migration, invasion, angiogenesis, and anticancer-drug resistance [13]. CD163, TGF-β, CCL2, and CCL5 are known as TAM biomarkers in lung cancer. TAMs constitute the predominant cellular component in the TME of lung cancer [14]. They not only act as immunosuppressive cells enabling immune evasion of NSCLC but also directly contribute to cancer cell proliferation, survival, invasion, and metastasis. TAMs induce tumor cell progression, resulting in poor prognosis for patients, and for NSCLC patients in particular [15].

The interleukin family (IL) influences metastasis and growth of lung cancer. It has been found that the expression of IL-10 correlates with the tumor diameter of NSCLC patients [16]. In addition, studies have shown that IL-6 and TNF-α promote metastasis of NSCLC by inducing epithelial-mesenchymal transition (EMT) [17]. The IL-1 family includes IL-1β, which induces cancer migration and invasion, promotes cancer growth and metastasis, and is secreted by TAMs, fibroblasts, immune cells, and cancer cells [18–20]. Immune-cell production of IL-1β has been widely studied [21,22]. IL-1β affects EMT, the basis of migration and invasion in several carcinomas [23–25]. In NSCLC, an IL 1β-treated group showed increased EMT along with high expression of the transcription factor SLUG, which is required for EMT [26,27]. In addition, high expression of IL-1β is associated with low survival rates in lung cancer patients [28,29]. In mice, IL-1R deficiency, which binds IL-1β, reduces prostate cancer cell growth and metastasis [30].

Cytokines play important roles in inducing cancer cell migration and invasion. Those that participate in migration and invasion-related signal transduction pathways include p21-activated kinase 1 (PAK1), extracellular signal-regulated kinase [31], c-Jun N-terminal kinase, p38, nuclear factor kappa B (NF-κB), and AKT serine/threonine kinase (AKT) [18,20,32–34]. PAK1, downstream of Rac1 and cdc42 in the Rho family, induces actin polymerization to regulate basic cell migration [31,35] and is associated with healthy cells as well as with cancer growth, migration, and invasion [35–37].

Tonicity-responsive enhancer-binding protein (TonEBP), also known as NFAT5, is a transcription factor in the Rel family of NF-κB and NFAT1-4 proteins [38–41]. It participates in regulating cellular homeostasis in relation to osmotic pressure, its expression is elevated under inflammation, and it is associated with cancer growth [41–43]. TonEBP participates in regulating cancer; clinical studies have revealed that high TonEBP expression increases mortality rates in NSCLC [39]. TonEBP induces hepatocellular carcinogenesis and is associated with liver cancer cell metastasis [42,43]. Although TonEBP is known to affect cancer migration in various carcinomas, the mechanisms by which it increases migration in lung cancer remain unclear. Therefore, in this study, we aimed to elucidate the mechanisms by which IL-1β and TonEBP affect cancer cell migration and invasion.

## Materials and Methods

### Chemicals and antibodies

Phorbol 12-myristate 13-acetate (PMA), Hoechst 33342, and IPA3 were purchased from Sigma-Aldrich (St. Louis, MI); PD 98059, SP 600125, and IKK2 inhibitor IV were purchased from Calbiochem (San Diego, CA); antibodies against PAK1, phospho-PAK1, extracellular signal-regulated kinase (ERK), phospho-ERK, p38, phospho-p38, IκBα, pre-IL-1β, and glyceraldehyde 3-phosphate dehydrogenase (GAPDH) were purchased from Cell Signaling Technology (Danvers, MA); and secondary anti-rabbit antibodies were purchased from Invitrogen (Carlsbad, CA).

### Cell culture

The human lung epithelial cell line A549 (CCL-185™) was purchased from ATCC (Manassas, VA, USA), and the human monocyte cell line THP-1 (40202) was purchased from Korean Cell Line Bank (Seoul, South Korea). A549 cells were cultured in RPMI 1640 medium (Welgene Inc., Daegu, South Korea) supplemented with 10% fetal bovine serum (GE Healthcare Life Sciences, Victoria, Australia), 50 U/mL penicillin, and 50 μg/mL streptomycin (Life Technologies, Carlsbad, CA), at 37°C in an incubator with 5% CO_2_. THP-1 cells were cultured in RPMI 1640 medium supplemented with 10% fetal bovine serum, as described above.

### A549 cocultured-MΦ conditioned media

THP-1 cells were differentiated into macrophages by treatment with 30 nM PMA in independent wells in the upper Falcon® Permeable Support for 6-well plate with a 1.0-µm Transparent PET Membrane (Corning, NY, USA), and A549 cells were cultured separately. After 24 h, the macrophage-differentiated upper insert was transferred to the wells with A549 cells, followed by 24 h of incubation. The upper insert containing the A549 cocultured-MΦ was cultured in fresh culture medium for 24 h, and the culture supernatant was collected following centrifugation of the A549 cocultured-MΦ-conditioned medium. IL-1β levels (in pg/mL) in the culture media were determined using the Human IL-1β Quantikine enzyme-linked immunosorbent assay (ELISA) Kit (R&D Systems, Minneapolis, MN).

### Transfection

Small interfering RNA (siRNA) for TonEBP or negative control was purchased from Integrated DNA Technologies (pre-designed DsiRNA, Coralville, IA). A549 cells were transfected using Lipofectamine 2000 reagent (Invitrogen, Carlsbad, CA) according to the manufacturer’s instructions.

### Immunoblotting

Cells were lysed using radioimmunoprecipitation buffer containing protease and phosphatase inhibitors. Total cell lysates were centrifuged at 12,000 × g for 15 min at 4°C, and the supernatants were collected. The protein concentration was determined using the SMART™ BCA Protein Assay Kit (iNtRON Biotechnology, Seongnam, South Korea). Proteins were separated via sodium dodecyl sulfate–polyacrylamide gel electrophoresis and then transferred to a nitrocellulose membrane. Nonspecific binding was performed via blocking with 5% non-fat milk and 5% bovine serum albumin in tris-buffered saline with Tween 20 (TBST; LPS solution, Daejeon, South Korea) for 30 min at room temperature. The membrane was incubated with primary antibodies in blocking solution overnight at 4°C, washed four times with TBST for 5 min each time, and then incubated with secondary antibodies. After washing with TBST, antibody-reactive proteins were detected using a chemiluminescence assay kit (Amersham Pharmacia Biotech, Freiburg, Germany).

### Migration and invasion assay

#### Wound healing assay

For migration or invasion experiments, 4 × 104 cells/well were placed in a 24-well plate and treated with conditioned medium (CM) or IL-1β for 24 h. In the case of invasion, the cells were separated and coated with Matrigel (Corning, NY, USA), diluted in the medium at the same time. After 24 h, the cells were scraped with a micropipette tip, washed with Dulbecco’s phosphate-buffered saline, and the medium was replaced. Cell images were taken after 24 h using a Nikon Eclipse Ti microscope (100× magnification, Nikon, Tokyo, Japan).

#### Transwell migration

For migration or invasion experiments, cells were split into 6-well plates and treated with CM or IL-1β for 24 h. Thereafter, the cells diluted with serum-free medium were split 1 × 10^4^/well in an 8-μm-pore inserts chamber (Corning, NY, USA) of 24 wells. In the case of invasion, 50 µL of Matrigel diluted with serum-free medium was especially coated before splitting the cells. The bottom wells of the inserts were filled with 10% a lung medium and incubated for 24 h. After incubation, the insert was fixed with 4% paraformaldehyde and then DAPI-stained with Hoechst 33342. Images were captured using a Nikon Eclipse Ti microscope (40× magnification, Nikon, Tokyo, Japan) and analyzed using the ImageJ software.

### Quantitative RT-PCR

Total RNA was isolated using TRIzol reagent (Life Technologies). Complementary DNA was synthesized from total RNA using the CellScript™ cDNA Master Mix (CellSafe, Yongin, South Korea). Relative RNA expression was detected via real-time PCR, using the Prism 7000 Sequence Detection System (Applied Biosystems, Foster City, CA) with TOPreal™ qPCR 2X PreMIX (Enzynomics, Daejeon, South Korea). The reaction conditions were as follows: 10 min at 95°C, followed by 40 cycles of 15 s at 95°C, 20 s at 60°C, and 30 s at 72°C. Primers (from Bioneer, Daejeon, South Korea) were as follows: for human GAPDH, forward, 5′-ACATCGCTCAGACACCATG-3′, and reverse, 5′-TG TAG TTG AGG TCA ATG AAG-3′; for human IL-1β, forward, 5′-ATGATGGCTTATTACAGTGGCAA-3′ and reverse, 5′-GTCGGAGATTCGTAGCTGGA-3′; for human PAK1 forward, 5′-CAACTCGGGACGTGGCTAC-3′, and reverse, 5′-CAGTATTCCGGGTCAAAGCAT-3′. mRNA levels, relative to those of GAPDH, were calculated according to the comparative 2^−ΔΔCq^ method.

### ELISA

A549 cells were incubated with macrophage CM in 2 mL of medium for 12 h. The medium was changed after 12 h to obtain IL-1β secreted by pure A549 cells. IL-1β levels (in pg/mL) in the cell culture media were determined using the Human IL-1β Quantikine ELISA Kit (R&D Systems, Minneapolis, MN).

### Statistical analysis

Data are presented as the mean ± SEM. Statistical significance was determined using Student’s *t*-test and one-way analysis of variance multiple comparison test using GraphPad Prism version 8.0 (GraphPad Software, Inc., USA).

## Results

### A549 cocultured-MΦ-secreted IL-1β enhanced A549 cell migration and invasion

**Figure 1:**
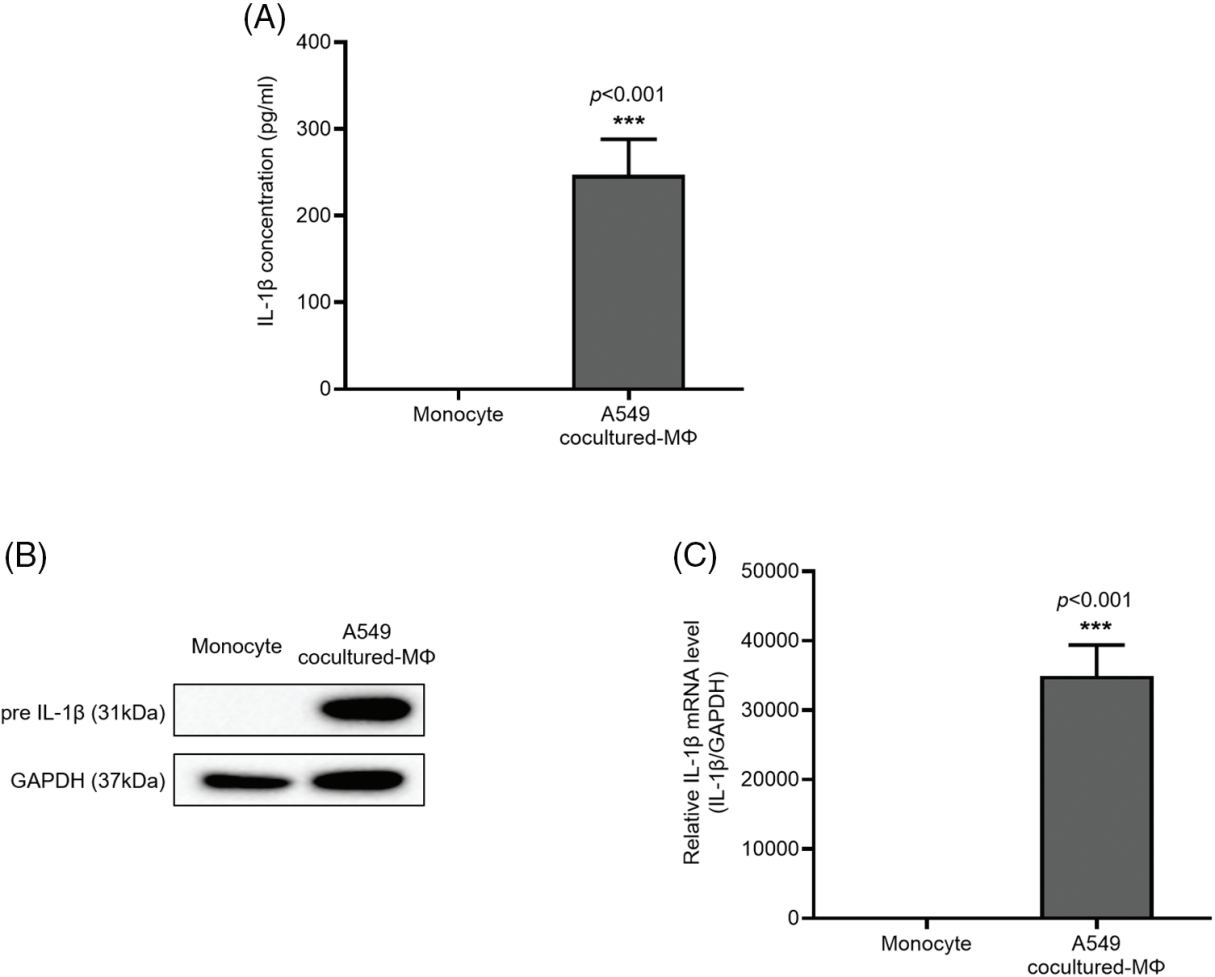
IL-1β secretion and expression in A549 cocultured-MΦ (A) IL-1β levels were determined via ELISA. (B) IL-1β and GAPDH expression were determined from whole-cell lysates. (C) IL-1β mRNA expression was analyzed via quantitative RT-PCR. Data are presented as the mean ± SEM of three independent experiments (***p < 0.001, compared with monocytes).

**Figure 2:**
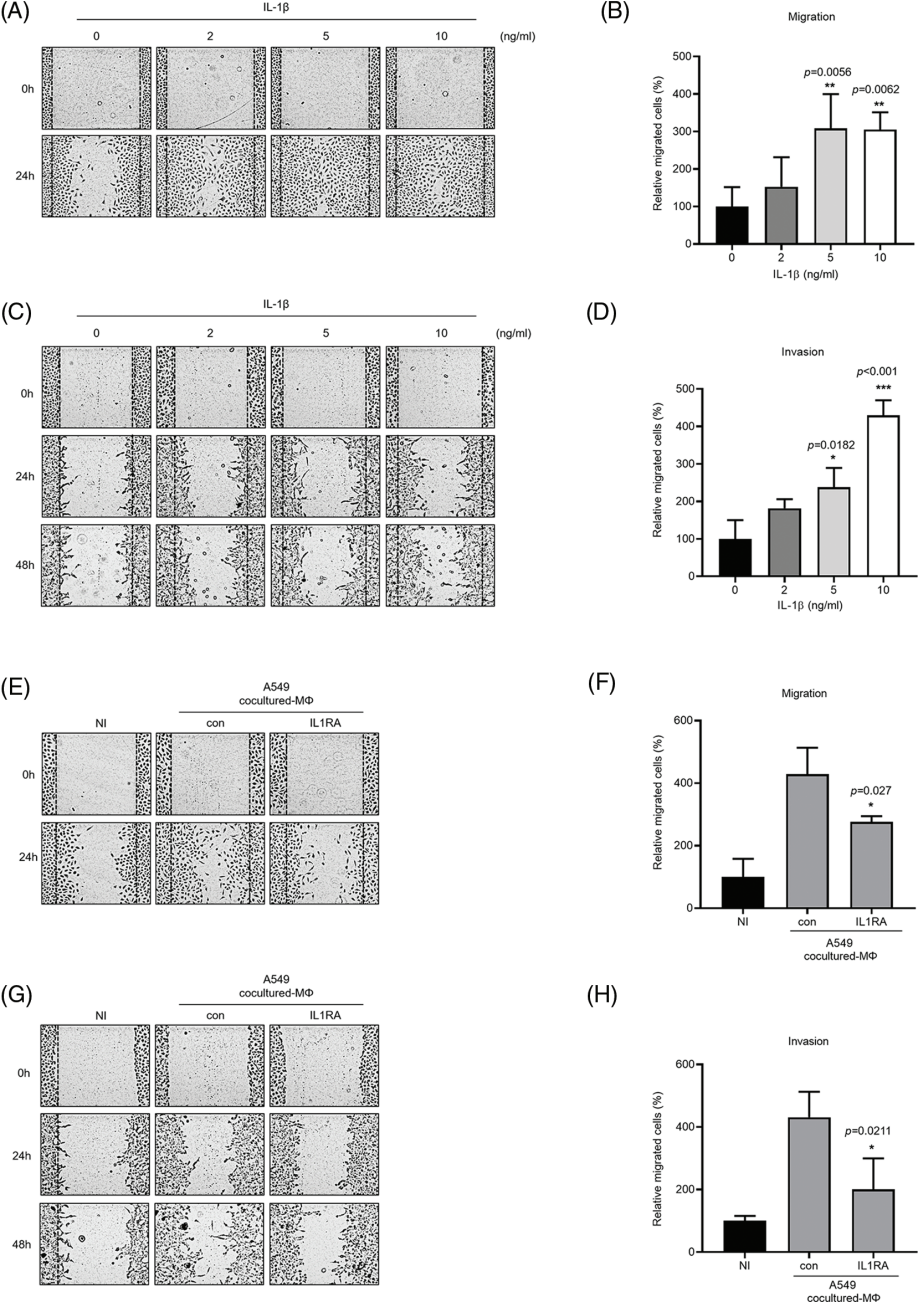
IL-1β secretion by A549 cocultured-MΦ promotes cancer cell migration and invasion. (A–D) A549 cells were treated with IL-1β. (E–H) A549 cells were incubated with A549 cocultured-MΦ-conditioned media in the presence or absence of IL-1RA for 24 h. Wound healing and transwell migration assays were conducted to determine the effects of IL-1β on cancer cell migration and invasion. Data are presented as the mean ± SEM of three independent experiments (*p < 0.05, **p < 0.01, ***p < 0.001, compared with 0 ng/mL and control; NI: non-induced).

IL-1β is secreted by macrophages and is known to be associated with migration and invasion of cancer cells. We confirmed that IL-1β was actually secreted *in vitro* (data not shown). We investigated whether IL-1β was secreted from A549 cocultured-MΦ and whether it induced migration and invasion in A549 cells. ELISA confirmed IL-1β secretion by A549 cocultured-MΦ (Fig. 1A). pre-IL-1β expression was higher in A549 cocultured-MΦ than in THP-1 monocytes (Figs. 1B and 1C). A549 cells treated with IL-1β showed concentration-dependent increases in cell migration and invasion (Figs. 2A–2D). To determine whether IL-1β affects the A549 cocultured-MΦ–induced increase in migration, we performed migration and invasion experiments by treating cells with an IL-1 receptor antagonist (Figs. 2E–2H). Under cotreatment with A549 cocultured-MΦ and the IL-1 receptor antagonist, migration and invasion were lower than when A549 cocultured-MΦ were applied alone. This suggests that IL-1β plays a key role in the A549 cocultured-MΦ–induced increase in migration and invasion.

### ERK, p38, and NF-κB inhibitors reduced IL-1β–induced migration and invasion

**Figure 3:**
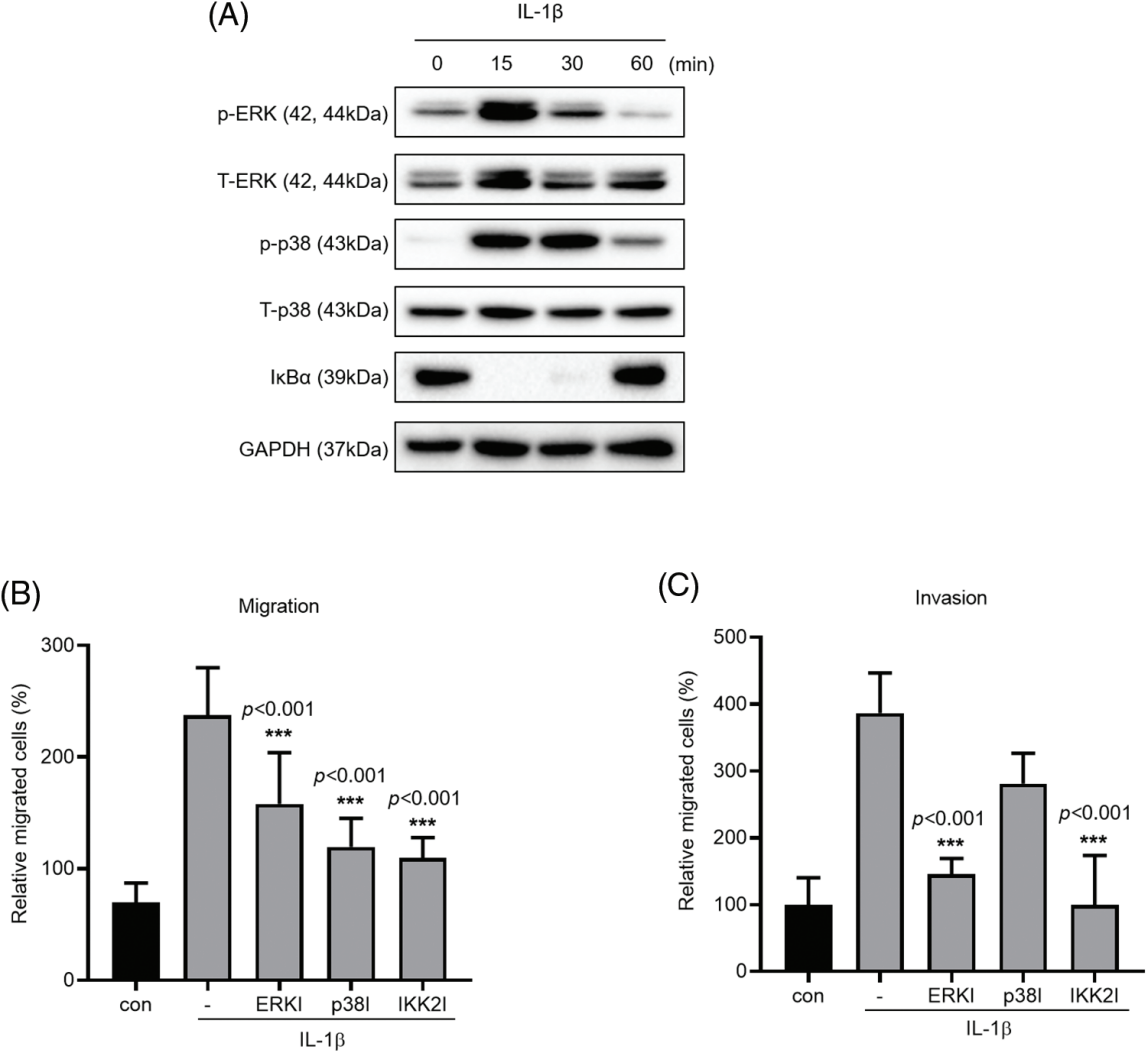
Effects of MAPK and NF-κB inhibitors on IL-1β–induced cancer cell migration and invasion. (A) p-p38/t-p38, p-ERK/t-ERK, and IκBα expression levels were determined using whole-cell lysates. (B, C) A549 cells were pretreated with ERK, p38 and IKK2 inhibitors for 1 h and then treated with IL-1β for 24 h. The effects of MAPK and NF-κB inhibitors on cancer cell migration and invasion were determined using a transwell migration assay. Data are presented as the mean ± SEM of three independent experiments (***p < 0.001, compared with the -).

The MAPK and NF-κB signaling pathways are known to induce IL-1β expression, which in turn promotes cancer cell migration and invasion. We verified this by treating A549 cells with IL-1β (Fig. 3A); this treatment induced ERK and p38 phosphorylation and reduced IκBα expression. To verify that these pathways affect cancer cell migration and invasion, we treated the cells with MAPK and IKK2 inhibitors (Figs. 3B and 3C); treatment with ERK, p38, and IKK2 inhibitors significantly reduced cell migration. Cell invasion was reduced significantly following treatment with ERK and IKK2 inhibitors but non-significantly following treatment with p38 inhibitor.

### PAK1 inhibitors reduced IL-1β–induced cancer cell migration and invasion

**Figure 4:**
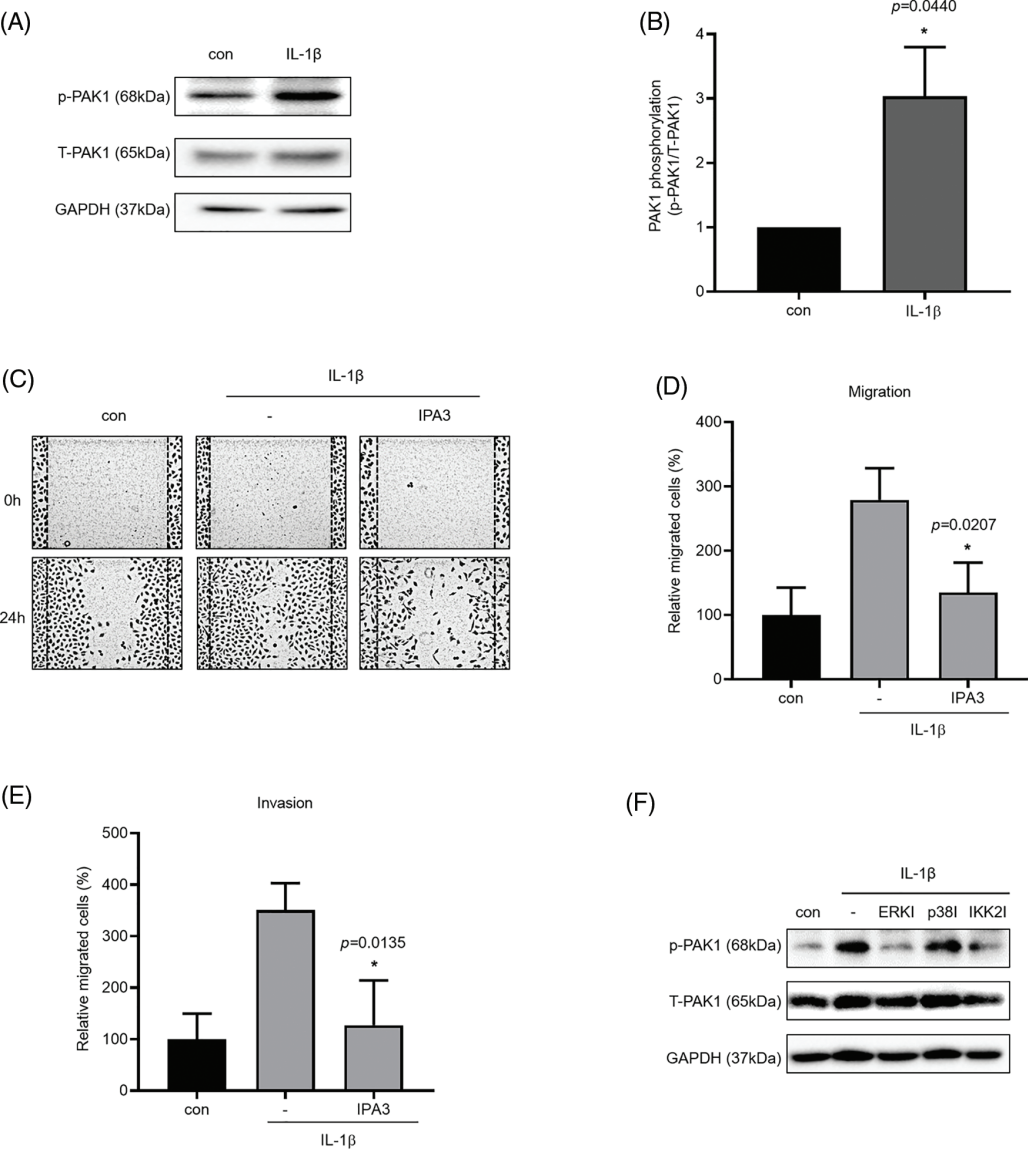
Inhibitory effects of IPA3 on IL-1β–induced migration and invasion of A549 cells. p-PAK1/t-PAK1 expression levels were determined using whole-cell lysates. (A, B) Western blot results. (C–E) A549 cells were incubated with IL-1β in the presence or absence of IPA3 for 24 h. The effects of IPA3 on cancer cell migration and invasion were determined via wound healing and transwell migration assays. (F) A549 cells were pretreated with ERK, p38 and IKK2 inhibitors for 1 h and then treated with IL-1β for 24 h. Data are presented as the mean ± SEM of three independent experiments (*p < 0.05, compared with the con and -; con: control).

We investigated the phosphorylation of PAK1, a signaling molecule that induces cancer cell migration. We examined whether PAK1 signaling participates in the induction of cancer cell migration and invasion by IL-1β. We found that IL-1β treatment increased p-PAK1 expression relative to the control (Figs. 4A and 4B). Treatment with IPA3, a PAK1-phosphorylation blocker, significantly reduced cancer cell migration and invasion (Figs. 4C–4E). Many papers have reported that PAK1 induces migration in cancer cells by regulating MAPK and NF-κB. However, other previous reports indicated that ERK2 regulates PAK1 [44]. To determine whether PAK1 signaling is related to MAPK and NF-κB, we investigated p-PAK1 expression following treatment with MAPK and IKK2 inhibitors; treatment with ERK, p38, and IKK2 inhibitors reduced p-PAK1 expression relative to the control (Fig. 4F). This suggests that IL-1β induces cancer cell migration and invasion via the PAK1 pathway, downstream of ERK, p38, and NF-κB signaling.

### TonEBP deficiency suppressed A549 cell migration and invasion

**Figure 5:**
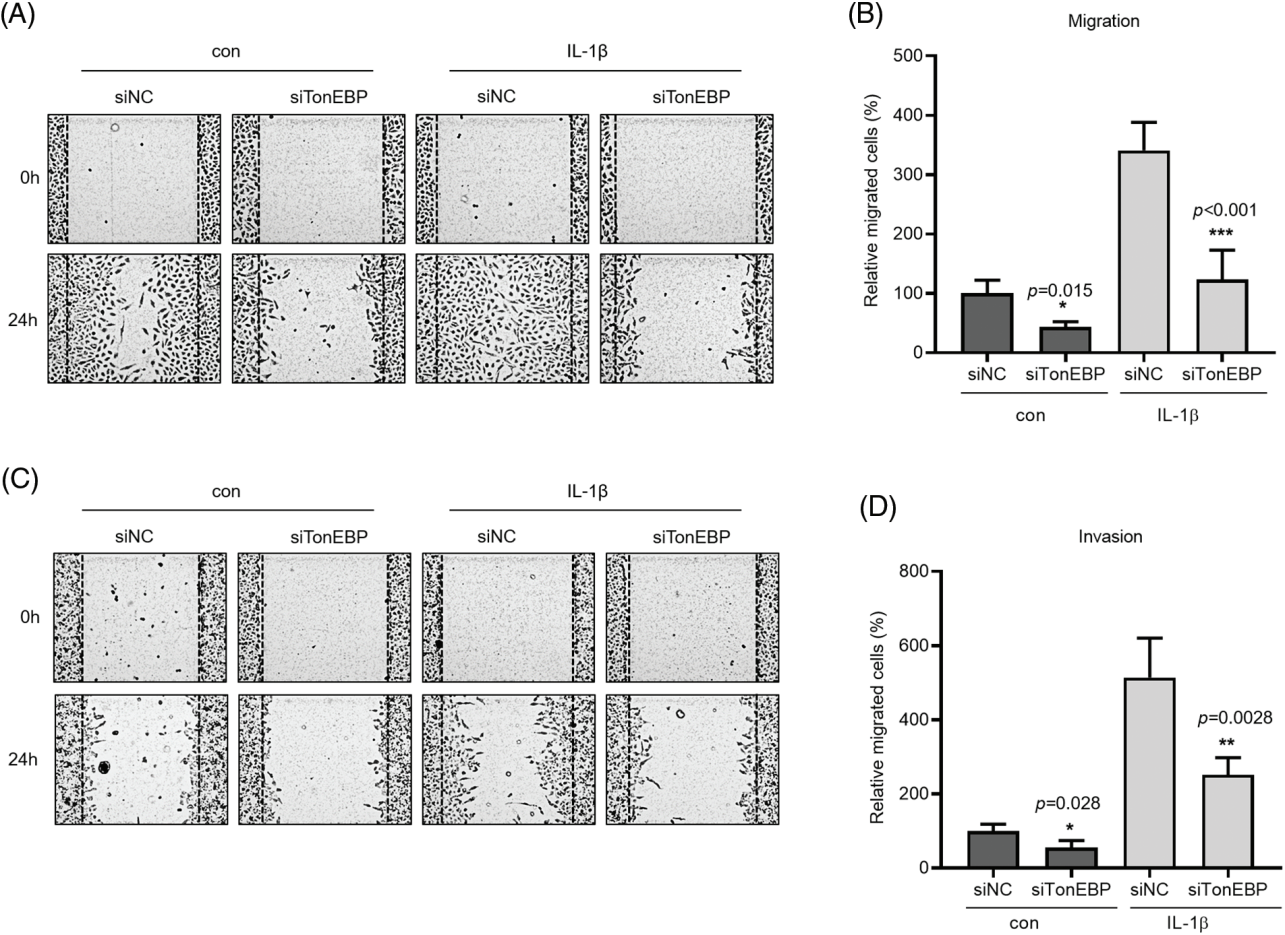
Effects of TonEBP depletion on cancer cell migration and invasion. A549 cells were transfected with siTonEBP or siNC for 24 h and then treated with IL-1β for 24 h. (A–D) Migration and Invasion were quantified via wound healing and transwell migration assays. Data are presented as the mean ± SEM of three independent experiments (*p < 0.05, **p < 0.01, ***p < 0.001, compared with the siNC treatment; NC: negative control).

TonEBP plays important roles in stress responses, osmotic regulation, and cancer, affecting cancer cell migration. We examined the association between TonEBP and IL-1β, both closely associated with cancer cell migration and invasion. TonEBP depletion via siRNA reduced cancer cell migration and invasion relative to the control (Figs. 5A–5D).

**Figure 6:**
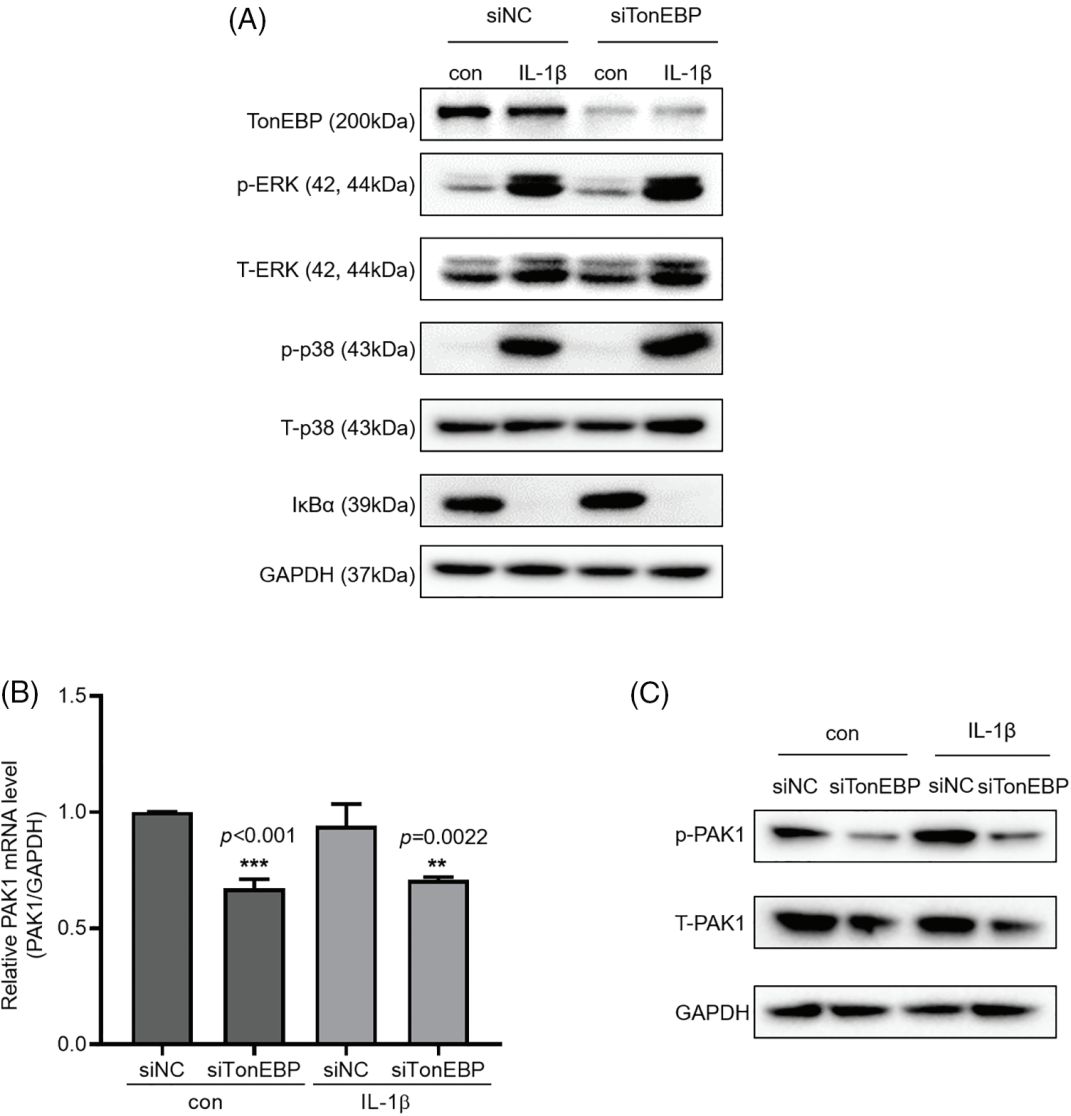
Expression of p-PAK1 and T-PAK1 on TonEBP deficiency. (A) TonEBP, p-p38/t-p38, p-ERK/t-ERK, and IκBα expression levels were determined using whole-cell lysates. A549 cells were transfected with siTonEBP or siNC for 48 h and then treated with IL-1β for 1 h. (B) PAK1 mRNA expression was analyzed via quantitative RT-PCR. (C) p-PAK1 and T-PAK1 expression was determined using whole-cell lysates. Data are presented as the mean ± SEM of three independent experiments (**p < 0.01, ***p < 0.001, compared with the siNC treatment; NC: negative control).

We then investigated how TonEBP regulates cell migration and invasion. To determine whether TonEBP affects IL-1β-activated ERK, p38, and NF-κB, we investigated ERK and p38 phosphorylation and IκBα expression following TonEBP depletion; we found that TonEBP depletion did not affect total or phosphorylated ERK, p38, and IκBα levels (Fig. 6A), but it reduced the protein and mRNA expression of PAK1, a signaling molecule downstream of MAPK and NF-κB, relative to the control (Figs. 6B and 6C). These results suggest that although TonEBP regulates PAK1 expression, it is independent of ERK, p38, and NF-κB signaling. Here, treatment with IL-1β did not directly increase TonEBP expression; however, depletion of TonEBP increased responsiveness to IL-1β–induced migration and invasion. In addition, TonEBP deficiency also affected the expression level of PAK1, indicating that the expression level of TonEBP is involved in IL-1β–induced migration and invasion as well as in the PAK1 signaling pathway.

## Discussion

Lung cancer is the cancer with the highest mortality rate worldwide. The high mortality rate of lung cancer can be attributed to difficulties in obtaining an early diagnosis and a high metastasis rate [1–3]. In general, the key to cancer metastasis is migration and invasion, and the TME plays an important role in these processes. For example, TAMs with high CD5L expression have been shown to be associated with poor prognosis in patients with papillary lung adenocarcinoma [45], and subtypes of high-grade serous ovarian cancer macrophages have been revealed to be associated with the prognosis of tumor extracellular matrix signatures [46]. Moreover, analysis of the TME in metastatic breast cancer identified several tumor-promoting genes in bone marrow cells and confirmed diversity in CAFs, endothelial cells, and mural cells [47]. It has been reported that TAMs create a type of cancer microenvironment that induces cancer metastasis and increases the migration and invasion of cancer cells [10].

**Figure 7:**
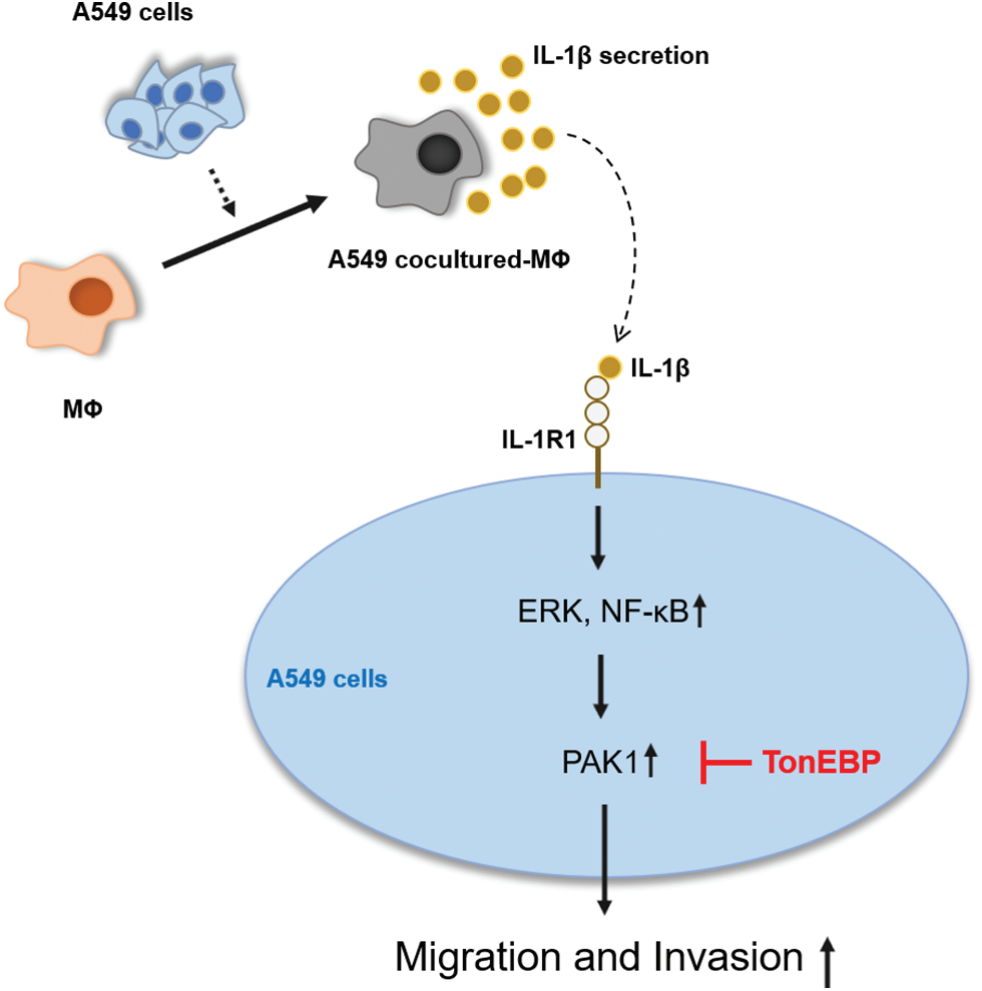
Schematic diagram of the effects of IL-1β and TonEBP expression on cell migration and invasion in A549 cells.

IL-1β and PAK1 are known to increase migration and invasion in various cancer cells, including lung cancer, which is consistent with our results. TonEBP has been reported to be related to migration and invasion in liver cancer cells; however, it is not known in other cancer cells, including lung cancer, and no association with IL-1β or PAK1 has been reported. Our results show that TonEBP plays an important role in IL-1β–induced cancer cell migration and invasion and that some of these effects proceed via the regulation of PAK1 expression in A549 cancer cells (Fig. 7).

The MAPK and NF-κB signaling pathways activate IL-1β–induced cancer cell migration and invasion, and inhibition of IL-1β–induced migration and invasion by an inhibitor or RNAi reduces cancer cell migration and invasion [48]. It has been reported that SCLC (Small cell lung cancer) and SLC (Squamous cell carcinoma) patient tissues have higher IL-1β expression than normal tissues [49]. We confirmed that migration and invasion induced by inhibitors of MAPK and NF-κB were reduced in the investigated lung cancer cell lines. This indicates that IL-1β induces cancer cell migration and invasion via the ERK and NF-κB signaling pathways.

PAK1 is known to be phosphorylated because of the binding of cdc42/rac1, a small GTPase protein, and the signal transduction of cdc42/rac1/PAK1 has been studied in tumors as well [50]. In esophageal squamous cell carcinoma, pharmacological inhibition of PAK1 reduces cancer cell migration and invasion as well as matrix metalloproteinase (MMP)-2 and MMP-9 expression [51]. In hepatocellular carcinoma, PAK1 is overexpressed together with Snail; further, PAK1 deficiency reduces cancer cell proliferation, migration, and invasion and induces apoptosis [37]. Here, we found that PAK1 plays a key role in the IL-1β–induced migration and invasion of lung cancer cells. PAK1 phosphorylation induced by IL-1β was reduced by inhibition of ERK and NF-κB. This suggests that IL-1β–induced ERK and NF-κB signaling induces cancer cell migration and invasion by regulating PAK1 phosphorylation. Previous studies have shown that A549 cells as well as various types of lung cancer cells induce migration and invasion [52,53]. This study has certain limitations. For instance, A549 cells harbor a k-RAS mutation and cannot be used to represent lung cancer cells with EGF mutations. Even in the case of THP-1 cells, artificially created conditions via *in vitro* co-culture with A549 cells cannot represent TAMs. To this end, it is necessary to isolate TAMs from lung cancer and conduct further research.

As a transcriptional regulator, TonEBP regulates various genes involved in specific tissues or diseases. TonEBP directly inhibits HO-1, a stress-inducing protein, in macrophages [54] and regulates inflammation-related TLR-induced genes by interacting with NF-κB [55]. TonEBP is essential in regulating osmotic pressure under cyclooxygenase-2–induced hyperosmotic conditions in cancer [56]. In addition, studies revealed that TonEBP promotes neuroinflammation and cognitive impairment via the upregulation of LCN2 in mice in a diabetes model [57]. It has been shown that TonEBP-p65 activates NF-κB by forming a complex with p300, a transcriptional coactivator. This suggests that TonEBP not only regulates genes directly but also indirectly [39]. Here, we found that TonEBP regulated PAK1. IL-1β-activated MAPK and NF-κB signaling were not affected by TonEBP deficiency, although it reduced the expression of PAK1, a downstream signaling molecule. Modulating the expression of TonEBP in A549 cells did not directly mediate IL-1β but altered the responsiveness of IL-1β to affect cancer cell migration and invasion. This suggests that TonEBP directly regulates PAK1 via the IL-1β–induced migration and invasion signaling pathways. To investigate the regulation of PAK1 expression by TonEBP, after depleting TonEBP, the expression of Rac1 and cdc42, which function upstream of PAK1, was examined; however, there was no significant difference (data not shown). TonEBP expression is associated with survival rate in lung cancer patients [39]. However, inhibitors of TonEBP have not yet been developed. Through this study, it was revealed that expression of TonEBP increases IL-1β reactivity. Therefore, future studies will investigate the inhibition effect of TonEBP expression on tumor size and metastasis using RNAi-nanoparticles in animal experiments. Animal experiments are expected to show the possibility of developing effective anticancer drugs using the regulation of TonEBP expression. Also, PAK1 reportedly affects the survival of PAK1-positive patients with NSCLC [58]. In an *in vivo* experiment, an inhibitor of PAK1 reduced the tumor size in a murine lung cancer model [59]. However, to the best of our knowledge, no clinical study has directly related lung cancer metastasis with TonEBP and PAK1 in patients with lung cancer, and this is an issue that warrants further investigation. Furthermore, the association between TonEBP and PAK1 is worth highlighting.

## Data Availability

The datasets used and/or analyzed during the current study are available from the corresponding author upon reasonable request.

## Abbreviations

NSCLC: Non-small cell lung cancer
TAMs: Tumor-associated macrophages
TME: Tumor microenvironment
MΦ: Macrophage
IL-1β: Interleaukine-1 beta
TonEBP: Tonicity-responsive enhancer-binding protein
PAK1: p21-activated kinase 1
JNK: c-Jun N-terminal kinase
NF-κB: Nuclear factor kappa B
